# Isolated Wrist Drop as the Sole Presentation of an Acute Embolic Ischemic Stroke

**DOI:** 10.7759/cureus.37404

**Published:** 2023-04-10

**Authors:** Maram Albandak, Mohammed Ayyad, Samah Abu Ajamia, Yousef M. N. Habes, Amjad Abdelalnbi

**Affiliations:** 1 Internal Medicine, Al-Quds University, Jerusalem, PSE; 2 Internal Medicin, Al-Quds University, Jerusalem, PSE; 3 Physiology, Al-Quds University, Jerusalem, PSE; 4 Internal Medicine, Al-Ahli Hospital, Hebron, PSE

**Keywords:** elderly, isolated wrist drop, watershed infarctions, rare presentation, ischemic stroke

## Abstract

Stroke is a major public health concern and a leading cause of morbidity and mortality worldwide. It often presents with a wide range of neurological deficits based on the neuroanatomical locus of the insult. Symptoms widely vary and usually occur in concordance with the distribution of the homunculus. Although rare, stroke can present with isolated wrist drop, which creates a diagnostic dilemma owing to the fact that this condition is by far more commonly caused by peripheral lesions. Moreover, localizing the site of injury is crucial for guiding therapeutic management and determining the overall prognosis of the condition. We present a 73-year-old patient with an isolated central wrist drop caused by an embolic ischemic stroke that was initially confused for a lower motor neuron pathology affecting the radial nerve.

## Introduction

Wrist drop is a medical condition commonly caused by peripheral lesions affecting the radial nerve and/or the brachial plexus. Although rare, central neurological pathologies can cause central wrist drop, which when isolated can be easily mistaken for a peripheral lesion [1]. Cortical wrist drop has many underlying etiologies that affect the central nervous system including trauma, tumors, and vascular insults [2,3]. Peripheral causes of wrist drop include humeral fractures, lead poisoning, and thiamine deficiency [4]. Importantly, the utilization of brain imaging and nerve conduction studies aids in the early characterization of the lesion and subsequent differentiation between central and peripheral causes. This is crucial as the recognition of the type and site of the lesion in a time-sensitive manner is pivotal for guiding management and determining the overall prognosis and morbidity of the primary insult. We herein report a rare case of isolated cortical wrist drop that was caused by acute embolic infarction in the contralateral cerebral hemisphere, which was initially confused with a peripheral etiology.

## Case presentation

A 73-year-old smoker gentleman was admitted to the medical unit in November of 2022 with weakness in the distal left upper limb presenting as an isolated wrist drop for the past few hours. The symptom occurred suddenly when he awoke from sleep and was associated with the perception of slightly slurred speech. Further investigation revealed no history of limb numbness, loss of consciousness, convulsions, altered level of consciousness, headache, blurred vision, or dysphagia. There was also no history of traumatic incidents or cervical disc herniation. On examination, he was conscious; alert; and oriented to time, place, person, and situation. Power and tone were normal in all limbs except in the distal left upper limb, where it was graded as 3/5 according to the Medical Research Council (MRC) Scale for distal extensor muscles. His physical exam was otherwise normal, including sensory and cranial nerves examination as well as systemic examinations. Moreover, the patient’s Glasgow Coma Scale (GCS) was 15/15 with negative meningeal irritation signs. He exhibited normal behavior on examination, which was verified by his family. No signs of ataxia or urinary incontinence were present. Importantly, the patient had a known case of long-standing uncontrolled hypertension diagnosed 15 years prior to his current presentation. He was also diagnosed with type two diabetes mellitus recently. He wasn't taking any regular medications at that time.

Vital signs were recorded upon admission and revealed a blood pressure of 133/71 mmHg, heart rate of 52 beats per minute, temperature of 36.5°C, and oxygen saturation (SpO_2_) of 92%. Electrocardiogram (ECG) was unremarkable. Laboratory investigations revealed mildly elevated lipids as well as minimally elevated levels of alanine aminotransferase (ALT), aspartate aminotransferase (AST), and C-reactive protein (CRP), as shown in Table 1. His complete blood count, kidney function tests, serum electrolytes, and alkaline phosphate were within the reference range. 

**Table 1:** Laboratory findings of the patient on admission *Serum electrolytes were within the normal range. ALT, alanine transaminase; AST, aspartate transaminase; LDL, low-density lipoprotein; HDL, high-density lipoprotein; LDH, lactate dehydrogenase; HbA1C, hemoglobin A1C; CRP, C-reactive protein; (↑): abnormally elevated level

Parameters*	Result	Normal value
ALT	59 ↑	0-45 U/L
AST	41 ↑	0-37 U/L
Alkaline phosphatase	108	Up to 115 U/L
Total bilirubin	0.5	Up to 1.2 mg/dL
Direct bilirubin	0.3	Less than 0.3 mg/dL
LDL	133 ↑	Moderate risk: 130-159 mg/dL
HDL	40	Moderate risk: 35-45 mg/dL
LDH	511 ↑	105-333 IU/L
Total cholesterol	192 ↑	Low risk: <200 mg/dL
HbA1C	7.1% ↑	Normal: <5.7%
Creatinine	1.32	0.5-1.5 mg/dL (modified for age)
Triglycerides	194 ↑	Moderate risk: 150-199 mg/dL
CRP	20 ↑	Up to 6 mg/L

Based on the patient’s presentation, a peripheral lesion of the radial nerve was suspected. Consequently, nerve conduction studies were ordered and were unrevealing. Additionally, a brain computed tomography (CT) scan was ordered and revealed a frontal midline iso-dense lesion with foci of calcification measuring 2.5×2.4 cm, associated with the right frontal lobe hypodense area suggesting a brain lesion with cytotoxic edema. There was no intracranial hemorrhage or major territorial infarction.

**Figure 1:**
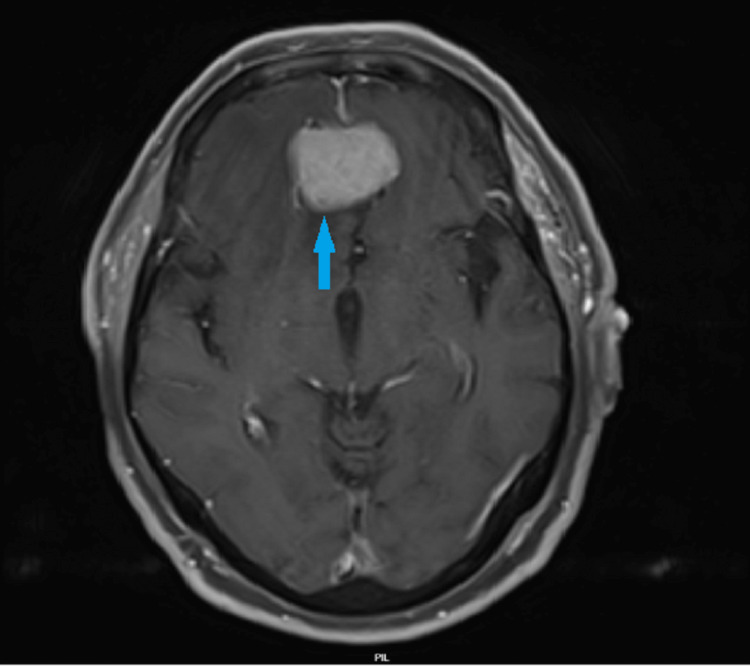
Brain MRI showing extra-axial dural-based solid mass suggestive of meningioma Brain MRI (T1-weighted imaging) showing an avidly enhancing extra-axial dural-based solid mass lesion (3.8×3.5×2.5 cm) in the anterior-inferior aspect of frontal lobes in the midline just above the cribriform plate (olfactory groove) suggestive of meningioma (blue arrow). MRI, magnetic resonance imaging

**Figure 2:**
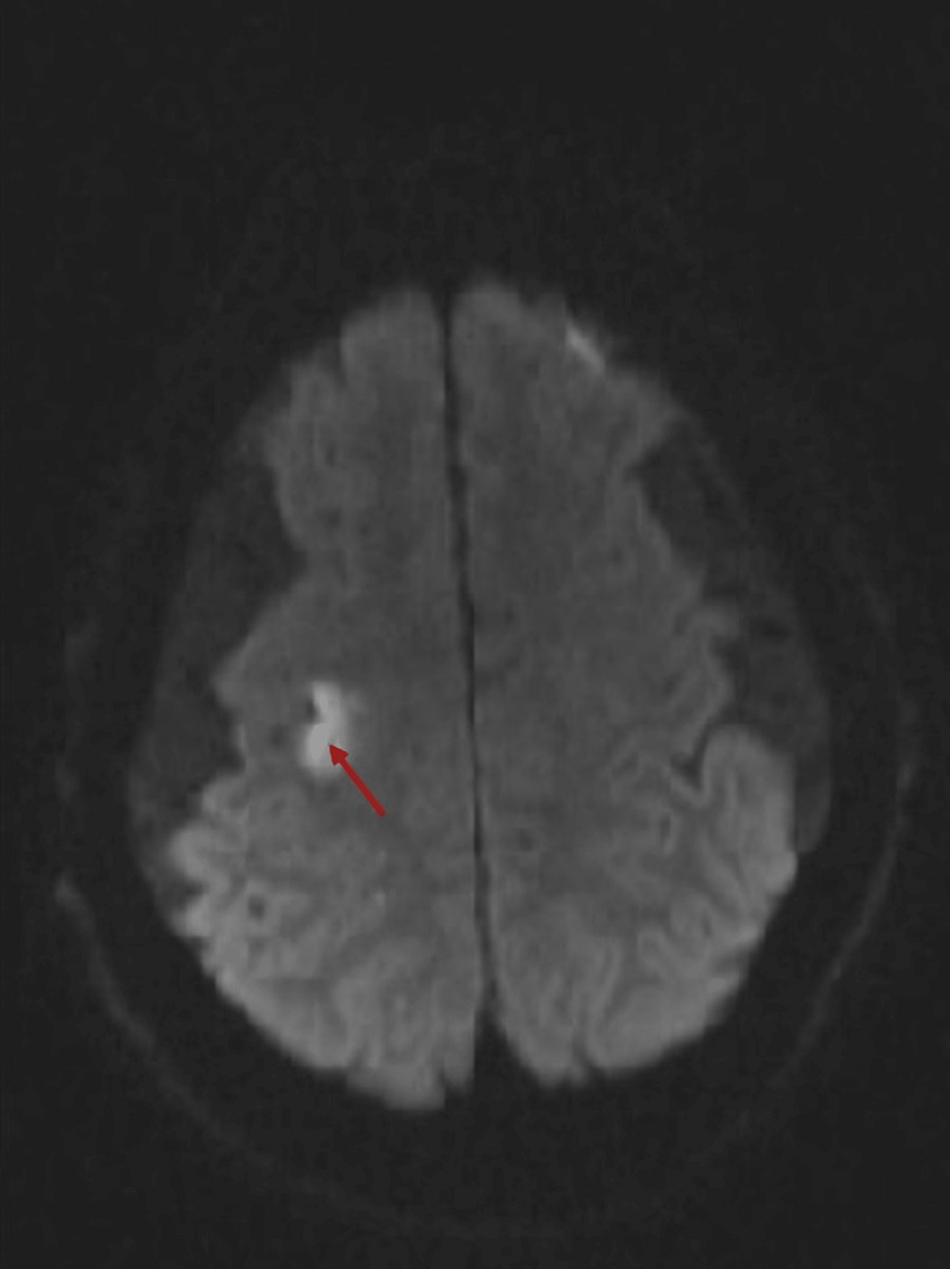
Brain MRI findings post-admission Diffusion-weighted MRI showing an acute infarction located in the “hand knob area” of the right precentral gyrus (red arrow). MRI, magnetic resonance imaging

Further brain imaging using magnetic resonance imaging (MRI) with and without contrast revealed an extra-axial dural-based solid oval mass measuring 3.8×3.5×2.5 cm. The mass was overlying the olfactory groove/planum sphenoidale and exhibited homogenously isointense signal on T1- and T2-weighted MRI. Moreover, it displayed avid intense homogenous enhancement following intravenous contrast administration (Figure 1). These findings were highly suggestive of olfactory groove meningioma. Additionally, diffusion-weighted MRI showed an acute infarction located in the “hand knob area” of the right precentral gyrus (Figure 2, red arrow).

 

Further investigation with carotid computed tomography angiography (CTA) revealed a soft plaque at the right carotid bulb and proximal right internal carotid artery, causing a 70% reduction in the luminal diameter of the vessel. Moreover, there was intimal thickening of the aortic arch and both common carotid arteries but none that caused significant vascular stenosis. Echocardiography and 48-hour Holter monitoring were also done to exclude cardiac causes of the patient’s symptoms, which were both normal and unrevealing.

Subsequently, the patient was treated with an ischemic stroke protocol including the administration of aspirin 300 mg/day, atorvastatin 80 mg/day, and clopidogrel 75 mg/day. Tissue plasminogen activator (tPA) was not administered due to the delayed presentation since his stroke happened the night before, as well as the presence of an intra-axial neoplasm imposing further limitation due to the increased risk of internal bleeding. He was kept under observation for three days and was then discharged in a stable general condition on an aspirin-extended-release dipyridamole combination (25 mg/200 mg twice a day) and referred for rehabilitation therapy in a specialized care center, with slow regain of his hand function. Furthermore, a consultation with a vascular surgeon was arranged for the patient for carotid endarterectomy evaluation of the right internal carotid artery.

## Discussion

Wrist drop refers to the inability to extend the wrist, which occurs due to weakness of the wrist extensor muscle groups innervated by the radial nerve or its branches. Most cases develop due to peripheral neurovascular, neuromuscular, and musculoskeletal causes; however, rare causes of central wrist drop have been reported in the literature [1,3-8].

Based on our patient’s initial presentation, a peripheral lesion of the radial nerve was suspected. However, sensory abnormalities were absent and nerve conduction studies were unrevealing. Given our patient’s risk factors, including old age, diabetes, and long-standing hypertension, central stroke was therefore suspected and included as part of our initial evaluation. A CT scan was done, and intracranial hemorrhage and major territorial infarction were excluded. An MRI was subsequently done and displayed evidence of an acute embolic stroke near the "hand knob" area, in addition to the presence of an incidental frontal meningioma. Considering the acute presentation and lack of any previous history of headaches, seizures, or focal neurological deficits, it was determined that the meningioma was unlikely to explain our patient’s wrist weakness. Meningiomas have rarely presented with transient ischemic attacks (TIAs) or strokes, but if they do, it occurs from the compression of the adjacent vasculature, which was not present in our case [9,10]. Additionally, the meningioma was located in the olfactory groove area, which is not responsible for hand movement. As illustrated in the literature, the hand knob area is located on the lateral surface of the contralateral cerebral convexity in the motor homunculus [11].

Interestingly, numerous cases of isolated wrist drop in the literature emerged from infarctions in varying vessels of the brain. Particularly, Timsit et al. reported six cases due to pseudo-ulnar palsy in patients with risk factors for carotid stenosis, such as smoking, hyperlipidemia, history of angina, and hypertension. The infarction was localized in the vascular border zones of the anterior cerebral artery (ACA), middle cerebral artery (MCA), posterior cerebral artery (PCA), and inferior parietal lobule [8]. Similarly, our patient had several atherosclerotic risk factors, in addition to the presence of 70% stenosis in the proximal right carotid artery, markedly contributing to stroke development. Hassan et al. described two cases of wrist drop due to posterior watershed infarctions in the MCA-PCA territory [6]. Takahashi et al. localized the hand area to be in the middle to lower part of the anterior wall of the central sulcus by investigating the symptoms of five patients presenting with weakness of the upper limb due to cortical infarction [7]. Collectively, the existing literature portrays a variable pattern in the etiology of central wrist drop, which physicians should be cognizant of.

While most ischemic lesions lie in the contralateral cerebral cortex, Venketasubramanian et al. reported a unique case of wrist drop due to a contralateral cerebral peduncle infarct [1]. Since the lesions present similarly and are hard to distinguish clinically, radiological imaging is crucial in localizing the site of the lesion of “cortical” wrist drop.

## Conclusions

Wrist drop is a medical condition caused by vast etiologies. Isolated cortical wrist drop, however, is a rare neurovascular disorder caused by disruption of the upper motor neuron pathways, with subsequent development of focal neurological deficits difficult to distinguish from peripheral causes. The differential diagnosis of isolated hand weakness is broad; nonetheless, the acute symptom onset and stuttering course of the condition, along with old age and the presence of cardiovascular risk factors, make ischemic stroke the more likely etiology. Brain imaging, nerve conduction studies, and physical examination findings are essential in distinguishing central from peripheral lesions. In particular, diffusion-weighted MRI is helpful for establishing an early diagnosis, as prompt aggressive management can prevent permanent deficits and improve overall prognosis. Therefore, physicians should have a high index of suspicion when presented with a case of isolated wrist drop in the appropriate patient population. 
